# Expression Atlas update: gene and protein expression in multiple species

**DOI:** 10.1093/nar/gkab1030

**Published:** 2021-11-24

**Authors:** Pablo Moreno, Silvie Fexova, Nancy George, Jonathan R Manning, Zhichiao Miao, Suhaib Mohammed, Alfonso Muñoz-Pomer, Anja Fullgrabe, Yalan Bi, Natassja Bush, Haider Iqbal, Upendra Kumbham, Andrey Solovyev, Lingyun Zhao, Ananth Prakash, David García-Seisdedos, Deepti J Kundu, Shengbo Wang, Mathias Walzer, Laura Clarke, David Osumi-Sutherland, Marcela Karey Tello-Ruiz, Sunita Kumari, Doreen Ware, Jana Eliasova, Mark J Arends, Martijn C Nawijn, Kerstin Meyer, Tony Burdett, John Marioni, Sarah Teichmann, Juan Antonio Vizcaíno, Alvis Brazma, Irene Papatheodorou

**Affiliations:** European Molecular Biology Laboratory, European Bioinformatics Institute, EMBL-EBI, Wellcome Genome Campus, Hinxton, UK; European Molecular Biology Laboratory, European Bioinformatics Institute, EMBL-EBI, Wellcome Genome Campus, Hinxton, UK; European Molecular Biology Laboratory, European Bioinformatics Institute, EMBL-EBI, Wellcome Genome Campus, Hinxton, UK; European Molecular Biology Laboratory, European Bioinformatics Institute, EMBL-EBI, Wellcome Genome Campus, Hinxton, UK; European Molecular Biology Laboratory, European Bioinformatics Institute, EMBL-EBI, Wellcome Genome Campus, Hinxton, UK; European Molecular Biology Laboratory, European Bioinformatics Institute, EMBL-EBI, Wellcome Genome Campus, Hinxton, UK; European Molecular Biology Laboratory, European Bioinformatics Institute, EMBL-EBI, Wellcome Genome Campus, Hinxton, UK; European Molecular Biology Laboratory, European Bioinformatics Institute, EMBL-EBI, Wellcome Genome Campus, Hinxton, UK; European Molecular Biology Laboratory, European Bioinformatics Institute, EMBL-EBI, Wellcome Genome Campus, Hinxton, UK; European Molecular Biology Laboratory, European Bioinformatics Institute, EMBL-EBI, Wellcome Genome Campus, Hinxton, UK; European Molecular Biology Laboratory, European Bioinformatics Institute, EMBL-EBI, Wellcome Genome Campus, Hinxton, UK; European Molecular Biology Laboratory, European Bioinformatics Institute, EMBL-EBI, Wellcome Genome Campus, Hinxton, UK; European Molecular Biology Laboratory, European Bioinformatics Institute, EMBL-EBI, Wellcome Genome Campus, Hinxton, UK; European Molecular Biology Laboratory, European Bioinformatics Institute, EMBL-EBI, Wellcome Genome Campus, Hinxton, UK; European Molecular Biology Laboratory, European Bioinformatics Institute, EMBL-EBI, Wellcome Genome Campus, Hinxton, UK; European Molecular Biology Laboratory, European Bioinformatics Institute, EMBL-EBI, Wellcome Genome Campus, Hinxton, UK; European Molecular Biology Laboratory, European Bioinformatics Institute, EMBL-EBI, Wellcome Genome Campus, Hinxton, UK; European Molecular Biology Laboratory, European Bioinformatics Institute, EMBL-EBI, Wellcome Genome Campus, Hinxton, UK; European Molecular Biology Laboratory, European Bioinformatics Institute, EMBL-EBI, Wellcome Genome Campus, Hinxton, UK; European Molecular Biology Laboratory, European Bioinformatics Institute, EMBL-EBI, Wellcome Genome Campus, Hinxton, UK; European Molecular Biology Laboratory, European Bioinformatics Institute, EMBL-EBI, Wellcome Genome Campus, Hinxton, UK; Cold Spring Harbor Laboratory, Cold Spring Harbor, NY 11724, USA; Cold Spring Harbor Laboratory, Cold Spring Harbor, NY 11724, USA; Cold Spring Harbor Laboratory, Cold Spring Harbor, NY 11724, USA; USDA ARS NEA, Plant Soil & Nutrition Laboratory Research Unit, Ithaca, NY 14853, USA; Wellcome Sanger Institute, Wellcome Genome Campus, Hinxton, UK; Edinburgh Pathology, University of Edinburgh, Institute of Genetics & Cancer, Edinburgh, UK; Department of Pathology and Medical Biology, GRIAC research institute, University of Groningen, University Medical Center Groningen, Groningen, Netherlands; Wellcome Sanger Institute, Wellcome Genome Campus, Hinxton, UK; European Molecular Biology Laboratory, European Bioinformatics Institute, EMBL-EBI, Wellcome Genome Campus, Hinxton, UK; European Molecular Biology Laboratory, European Bioinformatics Institute, EMBL-EBI, Wellcome Genome Campus, Hinxton, UK; Wellcome Sanger Institute, Wellcome Genome Campus, Hinxton, UK; European Molecular Biology Laboratory, European Bioinformatics Institute, EMBL-EBI, Wellcome Genome Campus, Hinxton, UK; European Molecular Biology Laboratory, European Bioinformatics Institute, EMBL-EBI, Wellcome Genome Campus, Hinxton, UK; European Molecular Biology Laboratory, European Bioinformatics Institute, EMBL-EBI, Wellcome Genome Campus, Hinxton, UK

## Abstract

The EMBL-EBI Expression Atlas is an added value knowledge base that enables researchers to answer the question of where (tissue, organism part, developmental stage, cell type) and under which conditions (disease, treatment, gender, etc) a gene or protein of interest is expressed. Expression Atlas brings together data from >4500 expression studies from >65 different species, across different conditions and tissues. It makes these data freely available in an easy to visualise form, after expert curation to accurately represent the intended experimental design, re-analysed via standardised pipelines that rely on open-source community developed tools. Each study's metadata are annotated using ontologies. The data are re-analyzed with the aim of reproducing the original conclusions of the underlying experiments. Expression Atlas is currently divided into Bulk Expression Atlas and Single Cell Expression Atlas. Expression Atlas contains data from differential studies (microarray and bulk RNA-Seq) and baseline studies (bulk RNA-Seq and proteomics), whereas Single Cell Expression Atlas is currently dedicated to Single Cell RNA-Sequencing (scRNA-Seq) studies. The resource has been in continuous development since 2009 and it is available at https://www.ebi.ac.uk/gxa.

## INTRODUCTION

Expression Atlas (https://www.ebi.ac.uk/gxa) is an added-value bioinformatics resource for gene and protein expression. It includes a database, user-interface and web-service that enables free access to information on gene expression across species, tissues, cells, diseases and other conditions. Expression Atlas was originally developed in 2009 to provide uniformly analysed microarray and bulk RNA sequencing (RNA-Seq) data from the archive ArrayExpress (1). Since then, Expression Atlas has grown and further developed to process selected datasets from NCBI’s Gene Expression Omnibus (GEO) (2), the European Nucleotide Archive (ENA) (3), controlled access datasets from archives such as dbGaP and the European Genome-Phenome Archive (EGA) (4). Since 2018, Expression Atlas has been extended to provide access to uniformly processed data from single-cell RNA-sequencing (scRNA-Seq) experiments within its component, the Single Cell Expression Atlas, described in our previous update in 2019 (5). Since then, the ArrayExpress archive is being superseded by the new multi-omics archive BioStudies (1) and our efforts focus on processing data sets directly from BioStudies.

With the continuing development of single-cell technologies and increasing expansion in data availability, scRNA-Seq datasets have become publicly available in larger numbers from a wider range of organisms. This has contributed to data from a wider selection of species in the Single Cell Expression Atlas. In particular, scRNA-Seq datasets from several plant species as well as data from cell atlas projects, such as Tabula Muris (6) are now available from Expression Atlas. The Human Cell Atlas (HCA) (7) has increased pace in generating datasets for different organs, which are regularly processed and included within the web-service of Expression Atlas. At the same time, bulk RNA-Seq datasets continued to grow, while recent, significant developments in metadata standards and analysis pipelines for mass-spectrometry proteomics datasets have rapidly accelerated the inclusion of proteomics datasets into the Expression Atlas.

In response to the COVID-19 pandemic and in collaboration with the effort of building the COVID Data Portal (8) at EMBL-EBI, we continue to source and uniformly analyse datasets that contribute to the research community's response to the COVID-19 pandemic. All these efforts have resulted in over 20% growth in the data volume in Expression Atlas during the last two years.

While the resource has significantly grown in data, the user interface of Single Cell Expression Atlas has also been improved for many different communities such as COVID, plants, HCA, mouse, and fly researchers. As datasets from many different human tissues are becoming available via the HCA, we have developed new features that enable an easy visualization of cell type specific gene expression. These include visual representations of 2D anatomy structures that can zoom into the tissue, down to the single-cell level, providing an anatomical representation of cells and making the results of human scRNA-Seq datasets easy to interpret by the research community.

## DATASETS AND SPECIES

**Figure 1.**
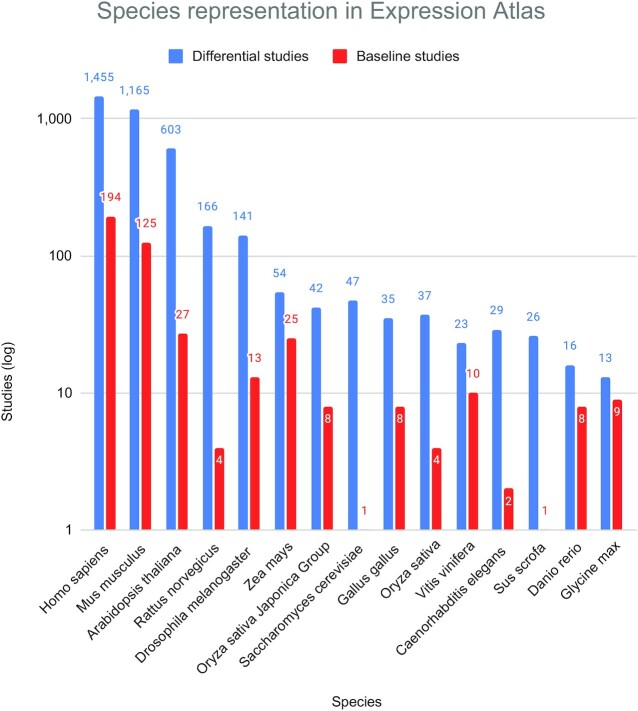
Top 15 most represented species in Expression Atlas, considering publicly available experiments across all technologies (RNA-Seq, Microarrays, Proteomics and Single Cell RNA-Seq), separated by differential and baseline studies. The 15 most represented species are shown, which jointly cover ∼94% of all studies. Separate varieties of *Oryza sativa* are considered, however when taken together they would make up for the second most represented plant species after *Arabidopsis thaliana*.

At the time of writing, taking together the Single Cell and bulk Expression Atlases cover data from 67 species through 4490 studies. This corresponds to an increase greater than 20% since the last update, where 89% (3982) are differential studies and the rest are baseline studies. All single-cell datasets are considered baseline. Figure 1 shows the list of the top 15 most represented species in all of Expression Atlas, separated by differential expression and baseline datasets. *Homo sapiens*, *Mus musculus* and *Arabidopsis thaliana* are the most represented organisms. Through all these studies, >4000 different ontology terms, from 29 different ontologies, are used to describe the experimental designs, cell types, tissue types and experimental conditions in Expression Atlas. The number of RNA-Seq assays in Expression Atlas has increased by >30%, while the number of cells in Single Cell Expression Atlas has increased by 650%, now exceeding 5.9 million cells. Among these ontologies, the most relevant ones in terms of usage in Atlas are the Experimental Factor Ontology (EFO) (9), Uber-anatomy ontology (UBERON) (10), Chemical Entities of Biological Interest ontology (ChEBI) (11), Cell Ontology (CL) (12), Cell Line Ontology (CLO) (13) and the Plant Ontology (PO) (14). Organisms are annotated using the NCBI Taxonomy (15).

### Datasets related to COVID-19 pandemic

Towards contributing to research during the COVID-19 pandemic, Expression Atlas added a collection of 22 single-cell studies and 25 bulk studies, relevant to this context. These studies were typically reutilising existing research data in order to elucidate the infection mechanisms or they were directly generated during the period of the pandemic and were rigorously prioritised for inclusion into the database. All the COVID-19 highlighted studies can be accessed either through the COVID-19 collections within both Atlases or through the EMBL-EBI COVID-19 Data Portal https://www.covid19dataportal.org/. All the studies placed in these collections were automatically indexed so that they are available as part of the main EMBL-EBI search. Importantly, selected relevant genes were linked from the EMBL-EBI COVID-19 Data portal to show their expression in Expression Atlas, beyond those selected studies to any study where they have expression, maximising access to expression data that was relevant for the study of the disease.

### Single cell expression atlas

At the time of writing, Single Cell Expression Atlas (the SC Atlas) includes 229 scRNA-Seq studies, spanning 18 species and above 5.9 million cells (a 6-fold increase since October 2019). The experiment designs metadata in the SC Atlas include manually curated annotations to >700 ontology terms across 19 different ontologies. The most relevant ontologies by usage are Cell Type Ontology (CL), Uber-anatomy (UBERON), Experimental Factor Ontology (EFO) and Plant Ontology (PO).

**Figure 2.**
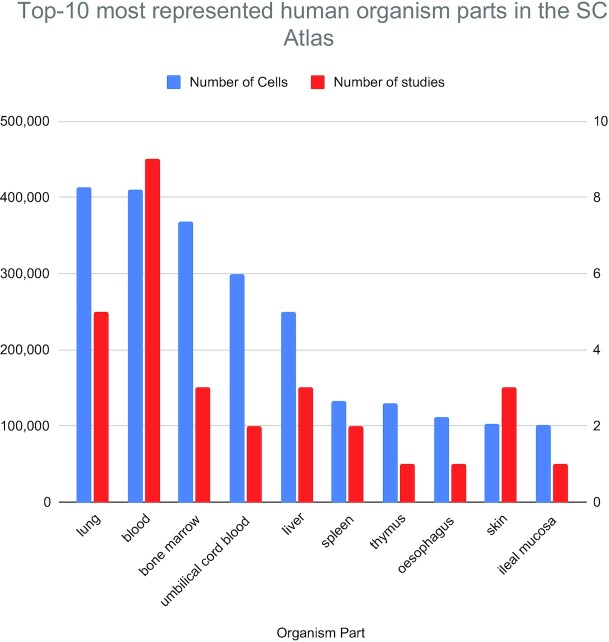
Top-10 represented human organism parts in Single Cell Expression Atlas, by number of cells (left) and number of studies (right).

The most represented species is human, with >3.5 million cells available through 103 studies. This is in part due to the involvement with the Human Cell Atlas (HCA) project; the SC Atlas provides visualization for eligible datasets from the HCA. Currently, there are 53 studies with >2.7 million cells that are shared between the HCA and the SC Atlas; these include original data generated by the HCA as well as selected studies imported from external resources and can be easily found in the SC Atlas under the featured Human Cell Atlas collection. Out of these, 31 studies have annotated cell types as provided by the authors of the studies, covering 232 different cell types annotated to ontologies. Human studies in the SC Atlas cover 49 different organism parts in humans. Figure 2 shows the top 10 most represented human organism parts in the SC Atlas, with lung and blood being the ones with more cells and studies. As part of the collaboration with the HCA, the SC Atlas will host all studies produced by the DiscovAIR project that centres on lung, and by the Gut Cell Atlas project, in addition to other HCA datasets.

The Human Cell Atlas has helped us to obtain user feedback which resulted in the wider prominence of author's cell types as the default view for cell clustering, the introduction of UMAP as an additional dimensionality reduction visualisation, and general improvements in the data analysis of the SC Atlas. This feedback has included constructive criticism from dataset author's when studies on the SC Atlas do not replicate their findings. Towards this we have made improvements in the analysis pipeline (details in the new functionality section), where the most relevant for this problem has been the introduction of optional batch correction for datasets that require it, based on their experimental design. The use of batch correction is decided on a dataset by dataset case by curators and bioinformaticians. While we recognise that the peer reviewed manual analysis and visualization done by authors will frequently result in the most correct scientific outputs, it is important as well to analyze datasets with a standardised pipeline, without any manual intervention, to understand how many of the conclusions hold in those conditions, both for reproducibility purposes and later dataset integrations with minimal differences due to analysis methods.

Among human expression datasets, 45 disease states are represented through >2.5 million cells in 78 studies. While most disease states are represented by a single study, there are some notable examples with more than a single study: type II diabetes mellitus (five studies), COVID-19 (four studies), lung adenocarcinoma (three studies), Parkinson's disease (two studies) and melanoma (two studies). Out of the 45 diseases, 19 are some form of cancer. Following *Homo sapiens*, the second most represented species is *Mus musculus*, with >1.3 million cells, comprising 71 studies, out of which 13 have cell type labels as inferred by authors, which include 180 different mouse cell types. These studies cover 51 different *M. musculus* organism parts and seven diseases (each disease with one study).

There are data from nine studies for fruit fly (*Drosophila melanogaster*, the main focus of Fly Cell Atlas), with >670 thousand different cells across 4 different organism parts, being the fourth most represented species within the SC Atlas. Out of these studies, three of them have authors inferred cell types annotated, providing overall data on 12 different cell types. The increased support for fruit fly, compared to a single study in October 2019, is part of an ongoing collaboration with the Fly Cell Atlas consortium at the data and ontologies level.

The most represented plant species is *A. thaliana*, which includes 12 studies, >670 thousand cells, 5 organism parts and 39 distinct author provided cell types, followed by *Zea mays* (>66k cells, two organism parts), *O. sativa* (>51k cells and two organism parts across two different varieties) and *Solanum lycopersicum* (>46k cells, one organism part and 10 distinct author provided cell types).

### Submissions handling

In order to integrate with these model organism communities, thought needed to be given to standardising metadata and raw/processed data requirements so datasets across multiple labs could be incorporated and compared across. Data integration and standardization must conform to the FAIR (16) principles to ensure this. A reproducible scRNA-Seq dataset comprises three components: raw data, processed data, and metadata (describing and linking to the raw data). This is implemented in our data submission tool, Annotare, which encourages users to submit raw/processed data and metadata via user-friendly templates, specific to their corresponding biological and technical requirements (17). The Human Cell Atlas Data Coordination Platform (HCA DCP, data.humancellatlas.org) and ENA data hubs are optimised to scale, to support high-throughput raw scRNA-seq data submissions (18).

Metadata describe the experiment, biological sample(s) and technical information about single-cell sequencing protocols that are essential for re-analysis. In particular, in partnership with the Gramene community (19), plant metadata templates have been designed to capture biological metadata. Considerations are also being made for other species-specific templates. For the relevant species, metadata is mapped to the closest species specific ontology (for plants Plant Ontology, for *Drosophila melanogaster* FlyBase FBbt (20)) term. For technological metadata—including library construction, cell isolation and cDNA amplification, new Minimum information for Single-Cell experiments (MinSCe) (21) standards have been developed. These terms have been incorporated into the Experimental Factor Ontology (EFO) with a unique label for each entity.

Submitted raw data and metadata are manually reviewed to ensure completeness and file integrity. Datasets are then stored in the ArrayExpress section in BioStudies. Each dataset is assigned a stable accession to reference in publications and to allow the work to be cited in accordance with FAIR principles (www.fairsharing.org). Raw data is brokered to the ENA, part of the International Nucleotide Sequence Database Collaboration (INSDC) (22), for secure, stable storage.

Unfortunately, many studies in the SC Atlas do not have the author's annotated inferred cell types. This illustrates the complexity of retrieving author's cell types annotations from the literature and deposited data that Atlas reprocesses, given the lack of a common standard to date to represent this metadata systematically in the community. It is imperative that the community agrees on standards for passing this information, as it is one of the most important findings in scRNA-Seq.

### Expression atlas: bulk transcriptomics and proteomics

**Figure 3.**
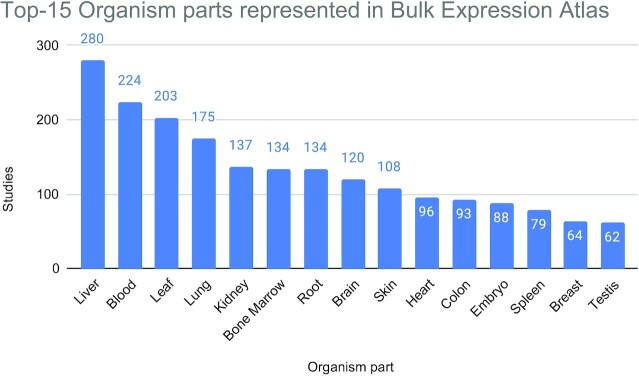
List of 15 organism parts with the highest number of studies in Bulk Expression Atlas. These 15 organism parts, across different organisms, cover a total of 1997 studies, which represents ∼62% of all studies that have an organism part annotation.

**Figure 4.**
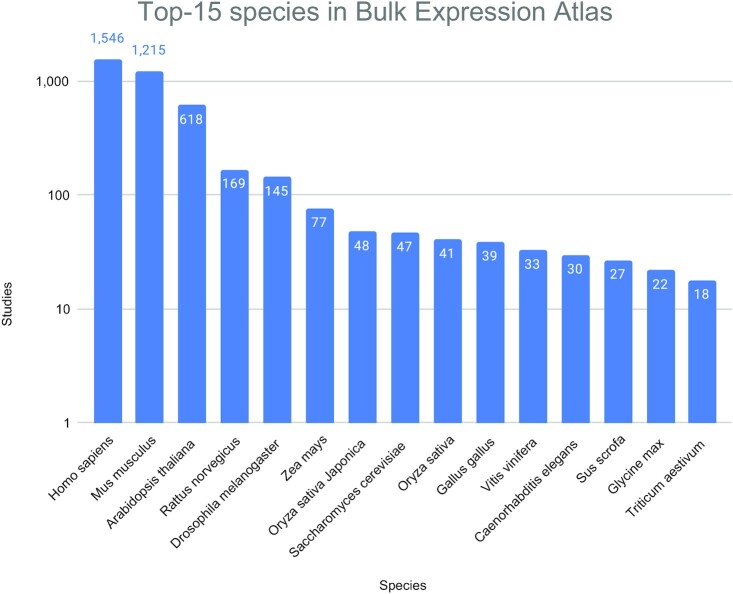
Top-15 most represented species in Expression Atlas bulk. These 15 species cover >95% of all studies in EA, where >50% of the studies are either Human or Mouse studies. Counting all three different varieties of rice (*Oryza sativa*) together, this species would be the second most represented plant species after *Arabidopsis thaliana*.

**Figure 5.**
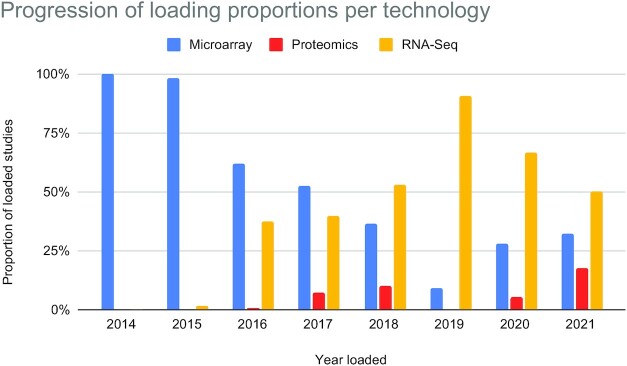
Proportion of studies loaded each year broken down by technology, for Expression Atlas bulk. Data for 2021 is incomplete due to pending loadings. Until 2019 included, there was a clear trend in the reduction of loading of Microarrays and an increase in loading of RNA-Seq studies.

At the time of writing, the bulk Expression Atlas (EA) comprises >4200 studies (>15 100 assays) across 65 different species. Out of these studies, >2900 are differential microarray studies, 1068 are RNA-Seq differential studies, 221 are RNA-Seq baseline studies and 58 are proteomics baseline studies. Through these studies, 784 different diseases, 991 different organism parts (Figure 3) and 638 different developmental stages are included, among other conditions and factors. While >65% of disease and organism part terms have ontology annotations, only ∼27% of developmental stages terms have ontology annotations, due to the complexity of their representation. Figure 4 shows the Top-15 most represented species in EA. Figure 5 shows how the proportion of studies of different technologies has changed over time for the past 7 years, where until 2019 included there was a tendency to reduce the proportion of microarray studies and increase the proportion of loaded RNA-Seq studies.

**Figure 6.**
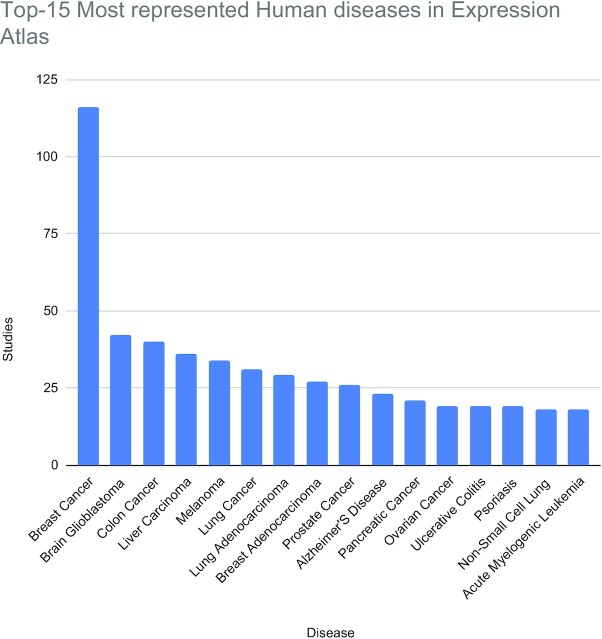
Most represented human diseases in Expression Atlas (bulk RNA-Seq, Microarrays and Proteomics) by number of public studies available. These diseases cover ∼47% of all the studies that have a disease annotation (1095), out of a total of ∼685 different human diseases annotated to all Atlas studies (this doesn’t account for diseases annotated at different granularity levels on the studies, for instance lung cancer and lung adenocarcinoma, which are counted separately).

As with single-cell data, *Homo sapiens* is the most represented species within EA, with >1500 studies. These studies include annotations to 685 different human diseases (in 1095 studies), 372 different human organism parts (through 1008 studies) and 75 different developmental stages (across 276 studies). Two thirds of all EA studies are disease related. Figure 6 shows a summary of the 15 most represented diseases in EA, where >80% of the highly represented diseases are some form of cancer.

### Proteomics

Since the last NAR update, we have continued to increase the content of proteomics datasets in Expression Atlas, working with the PRIDE (23) team at EMBL-EBI. EA now includes protein expression results coming from 59 proteomics datasets. The current datasets can be split into two main groups: Data Dependent Acquisition (DDA) and Data Independent Acquisition (DIA). DDA approaches have been the main ones used in proteomics. As such they are quite mature and rely on the selection of the most intense signals (corresponding to peptides) in the mass spectra, for identification and quantification purposes. On the other hand, DIA approaches have been established more recently and as such they are less mature and more complex. Their big advantage is that they are less biased in terms of the selection of peptides to be analysed, since all signals can potentially be considered. This decreases the amount of missing values when compared with DDA approaches.

Datasets generated using DDA approaches (49 datasets). In this case, MaxQuant (24) was used as the analysis software, followed by an in-house post-processing pipeline. A first group of datasets included cell-line and human tumour samples, which enabled us to generate an integrated landscape of protein expression in human cancer (25). Additionally, three groups of baseline tissue-based datasets (grouped per organs) are now available, coming from a wide variety of human (32 organs represented (26)), mouse (12 organs) and rat (8 organs) samples.

Datasets generated using DIA approaches (10 datasets at the time of writing). In this case, an in-house analysis pipeline was built using OpenSWATH as the base (https://github.com/PRIDE-reanalysis/DIA-reanalysis). These datasets constituted a pilot project to study the feasibility of performing a systematic reanalysis of DIA datasets and included cell-line, human cancer-related and plasma samples (27).

As a result of these efforts, EA users can now access increased proteomics expression information in the same interface as gene expression, providing an effective manner of transcriptomics and proteomics data integration. The current level of integration is possible because protein expression data is reported in a gene-centric manner.

## NEW FUNCTIONALITY

### Anatomograms in single cell expression atlas

**Figure 7.**
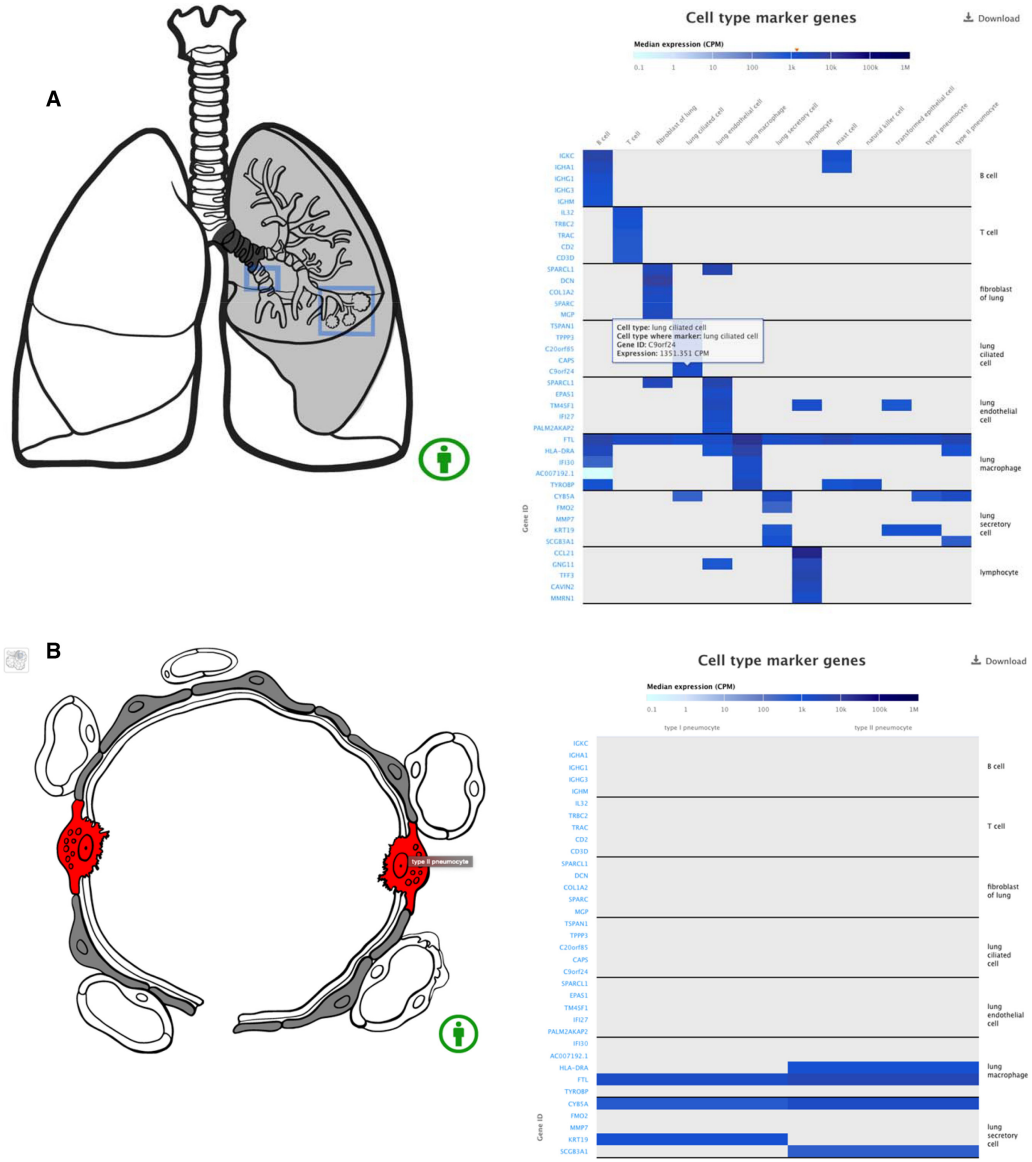
(**A**) The Single Cell Expression Atlas organ anatomogram for lung (for example shown at https://www.ebi.ac.uk/gxa/sc/experiments/E-GEOD-130148/results/anatomogram), displaying marker genes for the different lung cell types. Hovering over specific sections of the heatmap gives more details about the gene's expression. As the user clicks on an active section of the lung anatomogram, the heatmap to the right changes to display only cell types that exist under that specific part of the organ. (**B**) As the user dives into more and more detailed views, it will end up at a cellular view, where in this case type I and type II pneumocytes are shown.

In Single Cell Expression Atlas, users can explore the expression of a specific gene of interest across different species and experiments and the data points are presented in either a t-Distributed Stochastic Neighbor Embedding (t-SNE) (28) or Uniform Manifold Approximation and Project (UMAP) (29) plot which showcases the variability of gene expression at the single-cell level. However, it can be difficult for a user to fully relate data from t-SNE or UMAP plots (and the clusters shown there) to the real-life complexity of the biological tissues they represent, and to see the cells and organs behind the dots. For this reason, we have developed a new interactive data visualisation tool – the organ anatomograms. The anatomogram is an anatomy diagram of a human organ or a region within. It consists of a chain of interlinked interactive images that display an organ and its substructures in increasing levels of detail, all the way to the cellular level. Its individual component parts are annotated with ontology terms and the anatomogram pipeline matches these with the inferred cell type annotations in each dataset. The anatomogram leverages the ontology structure to also highlight corresponding parent structures in any of the higher-level images within the given organ anatomogram stack. This puts individual cell types identified through analysis of single-cell sequencing experiments in a broader structural context within each tissue/organ. Anatomograms also allow users to quickly discover top cell type markers for each cell type in an experiment. Figure 7 shows the lung anatomogram as an example, with the accompanying cell type and marker genes heatmap changing as the user goes from the high level organ view to the cell view. Currently, the SC Atlas has released anatomograms for lung, pancreas, placenta and liver with more anatomograms and more functionality linked to them on the way. The anatomograms pipeline has been designed to allow this feature to be embedded in third party websites and resources.

### Single cell visualization and data analysis improvements

**Figure 8.**
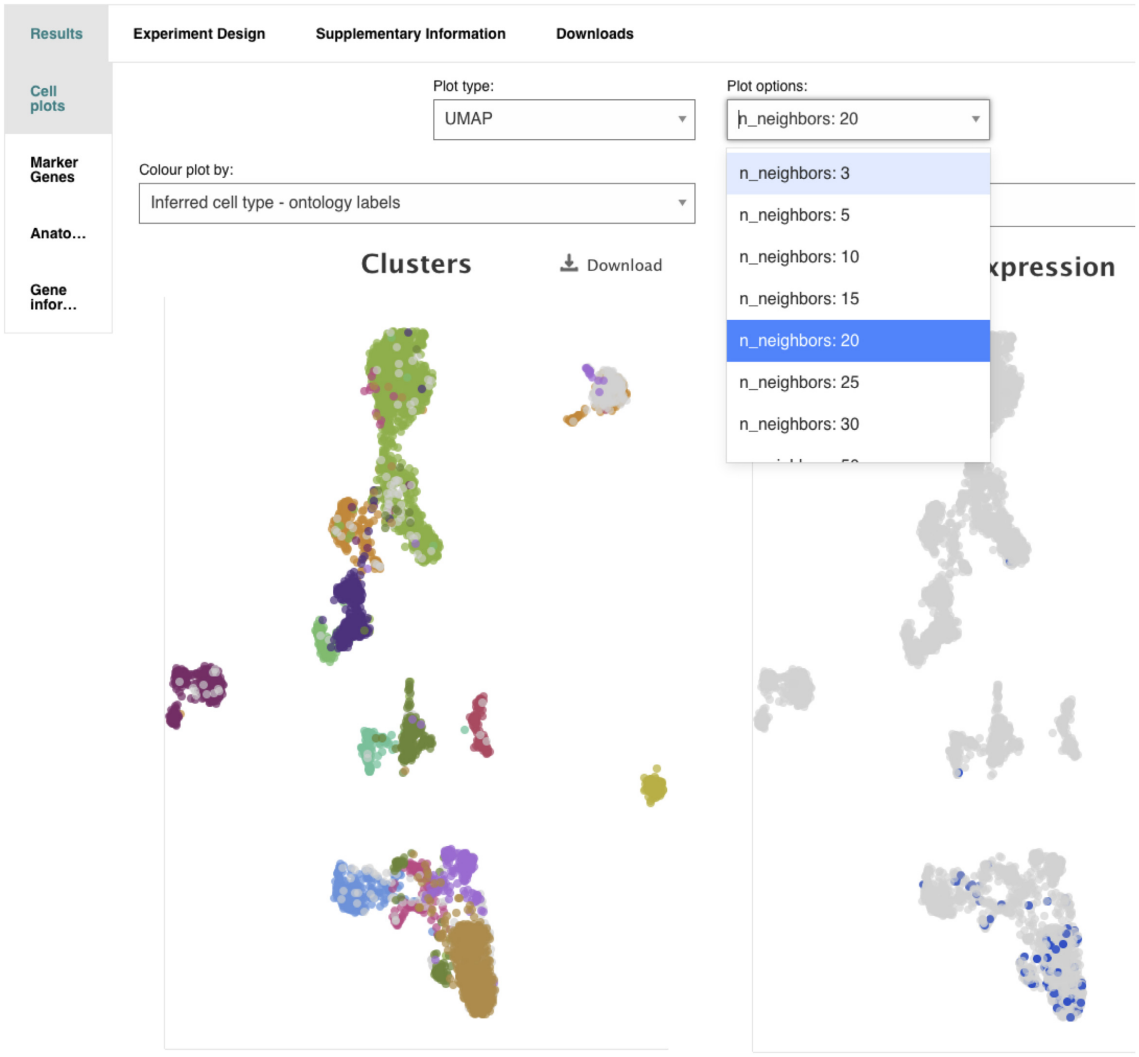
New selectors for dimensionality reduction cell plots, where the user can choose whether to use UMAP or t-SNE at different scales (plot options). By default, the landing page will show cell types as inferred by the author of the study if available (current field selected in ‘Colour plot by:’).

The current release of the SC Atlas provides visualisation of cell clusters, gene expressions levels and metadata fields values through t-SNEA and now as well UMAP dimensionality reduction methods, in both cases with predefined set of values for the most relevant scale parameters in each case (perplexity and number of neighbours, respectively). Figure 8 illustrates how the web UI allows the user to select the dimensionality reduction to use and the scale desired. By default the UI will display a UMAP layout of the cells on an intermediate scale level, showing by default the author's inferred cell types if available, or else Atlas calculated cluster annotation at an intermediate resolution value. Marker genes are now also calculated for the author's inferred cell types as well as for the SC Atlas calculated clusters.

In addition to dimensionality reduction plots, marker genes for Atlas calculated clusters can be seen through the marker genes heatmap. All dimensionality reduction plots (t-SNE and UMAPs for cell types, clusters, metadata values and specific gene expressions) as well as marker genes heatmaps can be downloaded from the UI, regardless of the size or number of points. Batch-correction in principal component space through Harmony (30) is applied during the analysis on selected experiments. This is done on the data that is used for dimensionality reduction views (t-SNE, UMAP).

The substantial increase in data volumes has required a number of improvements on the web application, database schemas, indexes and data analysis pipelines to maintain a quick analysis and fast access to the data on the web browser. In addition, downloads through the web UI in the form of zipped bundles have also been improved. Multiple experiments can be downloaded in a single archive by clicking the checkbox in the Download column, and this functionality works in conjunction with filters applied to experiments. Users can select during the download process whether they want data (matrices), metadata (annotations) or both.

Besides making text-format expression matrices, cell clusters and marker gene lists for each experiment available on the FTP (the link available from the SC Atlas interface) and through the SC Atlas web UI, the analysis pipelines now also generate an AnnData (31) file for each study (available at the same FTP address) with all cell groups (clusters and authors inferred cell types), dimensionality reduction embeddings (PCA, UMAP, tSNE) and marker genes for different cell groups to facilitate inspection and re-analysis of that data through environments such as Scanpy or visualization frameworks such cellxgene (32) or UCSC Cell Browser (33).

In response to requests from users, the resource has now the capacity to accept studies with an embargo period. This means that authors can deposit their data to be loaded on the SC Atlas and this will be kept private until the manuscript that explains the dataset is published. We have also created the Expression Atlas application ontology, built on-demand to maximise the search abilities of Expression Atlas. The Expression Atlas application ontology is constructed by the automated import of terms from a curated list of pre-selected ontologies such as UBERON, CL, Mondo Disease Ontology and FlyBase ontologies (FBbt and FBdv), among others. In addition, *de novo* terms are created and added as needed.

Single Cell Expression Atlas downstream analysis is run mostly through Scanpy, by using the Galaxy (34) tools, Bioconda (35) packages and Biocontainers (36) described for the SCiAp setup (37), which facilitates third party users to replicate both the individual tool and workflow. These tools can be easily installed on any Galaxy instance, but they are also freely available to use at https://humancellatlas.usegalaxy.eu/ Galaxy instance, where the four release versions of the SC Atlas downstream analysis workflows (which made possible the changes described in this section) are also prominently displayed and available for re-use.

## FUTURE DIRECTIONS

The development of an improved search functionality for Single Cell Expression Atlas is nearing completion and will be released shortly. Single Cell Expression Atlas will soon have the ability to search not only by genes of interest but also for specific conditions (diseases, developmental stage, cell type, organism part or any field encoded in the metadata of studies). This new search capability will lead to a holistic view of the data available for that condition, through the visualization of results in an interactive cell type wheel and an experiment - cell type heatmap.

Development of anatomograms joint views with other interactive elements is under way to connect anatomograms to other plots and tables within the experiment page as well as across experiments. More anatomograms will be released, as well as more experiments featuring the anatomograms.

A number of improvements are currently being tested for inclusion in future analysis pipelines, including multiplet removal, improved batch handling and filtering of cells with high mitochondrial content. A new cell typing functionality will be unveiled, which will enable users to approach Single Cell Expression Atlas with their cell expression counts matrices and get back potential cell types assignments for their cells in the organism of interest.

Other model organism single-cell datasets are still relatively rare compared to the volume of human and mouse datasets: as an example there are 27 single-cell datasets from *A. thaliana* versus 2511 from human and 3355 from mouse at NCBI GEO. Integrating these datasets into organism-wide cell atlases will help generate a more comprehensive understanding of organism genetics and enable further advancements in these fields that would not rely on insights from individual datasets, but consensus from multiple studies. Hence, it is essential for the datasets to be standardized for cross-comparison. As part of this integration, we have focused on two species communities: the plant genomics community, for plant datasets, in particular *A. thaliana* and the Fly Cell Atlas consortia.

Bulk EA will see a new view for summarising the expression of a gene in all organism parts (merging data across studies) for certain key organisms. This will have a differential view and a baseline view, where different experiments will be merged through meta-analysis and batch correction methods.

In the context of proteomics datasets, additional efforts will be put in re-analysing and representing differential datasets in the Expression Atlas interface. This is challenging, among other reasons, due to the downstream statistical analysis required, and also due to the limited sample metadata annotations of MS2-labelled datasets in PRIDE. Additionally, efforts need to go into the automatization of many of the steps in the re-analysis, post-processing, and incorporation of the results in Expression Atlas. Also as a key point, the Expression Atlas data model will be extended to improve the representation of protein entities.

## DATA AVAILABILITY

Expression Atlas and Single Cell Expression Atlas are available for users at https://www.ebi.ac.uk/gxa/ and at https://www.ebi.ac.uk/gxa/sc/ respectively. Expression Atlas web application is open source and available in the GitHub repositories https://github.com/ebi-gene-expression-group/atlas-web-single-cell and https://github.com/ebi-gene-expression-group/atlas-web-bulk among others.
